# 11g, a Potent Antifungal Candidate, Enhances *Candida albicans* Immunogenicity by Unmasking β-Glucan in Fungal Cell Wall

**DOI:** 10.3389/fmicb.2020.01324

**Published:** 2020-06-30

**Authors:** Xin Huang, Yu Liu, Tingjunhong Ni, Liping Li, Lan Yan, Maomao An, Dazhi Zhang, Yuanying Jiang

**Affiliations:** ^1^Department of Pharmacology, Shanghai Tenth People’s Hospital, Tongji University School of Medicine, Shanghai, China; ^2^School of Pharmacy, Second Military Medical University, Shanghai, China

**Keywords:** *Candida albicans*, antifungal agent, β-glucan, dectin-1, immunogenicity

## Abstract

In the course of optimizing GPI biosynthesis inhibitors, we designed and synthetized a 2-aminonicotinamide derivative named 11g. After evaluating the antifungal activity of compound 11g *in vitro*, we investigated the influences of 11g on fungi immunogenicity. In addition, we also took advantage of murine systemic candidiasis model to investigate the protective effects of 11g *in vivo*. Results show that 11g exhibited potent antifungal activity both *in vitro* and *in vivo*. Further study shows that 11g caused the unmasking of fungi β-glucan layer, leading to stronger immune responses in macrophages through Dectin-1. These results suggest that 11g is a very promising antifungal candidate, which assists in eliciting stronger immune responses to help host immune system disposing pathogens. The discovery of 11g might expand the toolbox of fungal infection treatment.

## Introduction

Fungi are encountered, ingested, and inhaled by human beings every day. As opportunistic pathogens, their infection spectrum ranges from benignly superficial colonization to systemic fungal infection. As a result of the increasing number of susceptible immunocompromised and immunosuppressed patients, the incidence rate of clinically relevant fungal infections has risen steadily in the past three to four decades (LeibundGut-Landmann et al., 2012; McLellan et al., 2012). *Candida* species, which caused mostly fungus-related morbidity and mortality, are the fourth leading cause of hospital-acquired bloodstream infections (Cowen et al., 2002). There are between 1.1 and 24 cases of candidemia per 100,000 individuals, killing more than 30% of their victims (Gudlaugsson et al., 2003; Wisplinghoff et al., 2004).

In response to this mounting therapeutic challenge, developing drugs available for human invasive fungal infections has been intensely spurred. Nowadays, there are four major classes of antifungals with different mechanisms widely used in clinic: azoles, polyenes, echinocandins, and fluorinated pyrimidines. However, their own limitations, such as drug resistance, narrow antifungal spectrum, adverse effects, variable pharmacokinetic, and limited types of formulation, etc., restrict their applications in clinic (Martin et al., 2003; Wisplinghoff et al., 2004; Pfaller et al., 2008, 2010; Baddley et al., 2010; Denning and Hope, 2010; Kontoyiannis et al., 2010). To date, systemic fungal infections are not only expensive to treat, but also extremely difficult to eradicate. Therefore, safe and effective antifungal candidates with novel mechanisms are urgently needed for the treatment of serious systemic fungal infections.

The cell wall is clearly of paramount importance to the survival of fungi, which are different from mammalian cells, and it is always taken into account when searching for new antifungal targets. Previous studies have shown that the *C. albicans* cell wall consists of two layers: The outer layer is enriched with mannoprotein, and the inner layer contains the skeletal polysaccharides β-1, 3-glucan and chitin (Gow et al., 2011). The most abundant cell wall proteins (CWPs) are covalently attached to the meshwork of inner layer through a glycosylphosphatidylinositol (GPI) remnant and β-1, 6-glucan, which are termed GPI-anchored cell wall proteins (GPI-CWPs) (Richard and Plaine, 2007; Chaffin, 2008). It has been reported that GPI-CWPs are responsible for a vast range of functions: They not only help maintain the integrity of fungal cell wall, but also play important roles in adhesion, filamentation, and sensing of the environment. What’s more, they also provide a heavily glycosylated and phosphorylated outer coat to shield fungi from host immune defense (Orlean and Menon, 2007; Klis et al., 2009). Therefore, as a promising antifungal target, GPI has been drawing attention of many researchers in recent years.

To date, several GPI biosynthesis inhibitors, such as BIQ, G884, 10b, and E1210, etc., have been synthetized and regarded as new antifungal agents (Supplementary Figure S1; Tsukahara et al., 2003; Nakamoto et al., 2010; Miyazaki et al., 2011; Mann et al., 2015). In the course of optimizing these compound structures, we designed and synthetized a 2-aminonicotinamide derivative named 11g (Ni et al., 2017). In 2017, our group reported that 11g could decrease the GPI content in the *C. albicans* cell wall (Ni et al., 2017). In this paper, we further investigated the antifungal effects and mechanisms of 11g, and then tested its protective effect in murine systemic candidiasis model. Our study shows that 11g exhibits a potent and broad-spectrum antifungal activity. In addition, we also demonstrate that 11g has protective effect *in vivo* through enhancing fungal immunogenicity and spurring host immune responses.

## Materials and Methods

### Antifungals

11g was synthesized by the Second Military Medical University (Shanghai, China). 11g and fluconazole (Sigma, St. Louis, MO, United States) were dissolved in dimethyl sulfoxide (DMSO; Sigma) and stored in a −40°C freezer until application. On the day of use, stored solutions were diluted with appropriate media to yield DMSO concentrations of 1%.

### Strains

All the strains used in this paper were provided by the Second Military Medical University and were maintained in 80% glycerol at −80°C.

### Mice

Female 6- to 8-week-old C57BL/6 mice were purchased from Shanghai Laboratory Animal Center of the Chinese Academy of Sciences (Shanghai, China). All mice were kept in barrier conditions at the Experimental Animals Center of Tongji University (Shanghai, China). License Number: SYXK (Shanghai) 2014-0026. All mouse experiments were done according to institutional guidelines and were approved by the Institutional Animal Use and Care Committee of Tongji University.

### Minimum Inhibitory Concentration (MIC) Determinations

Assays were performed according to the broth microdilution method detailed by the Clinical and Laboratory Standards Institute (CLSI) in documents M27-A3 (Ma et al., 2015). Briefly, the cell suspensions were diluted with RPMI 1640 medium (Invitrogen, Carlsbad, CA, United States) to obtain inoculum sizes of 1 × 10^3^ cells/mL. The initial concentrations of antifungal agents ranged from 0.0313 to 64 × 10^–3^ mg/mL. MIC_80_ values were read after 24 h at 30°C. The results were expressed as the median values of MIC_80_ from three independent experiments. The MIC_80_ value was defined as the lowest concentration, at which a prominent decrease (≥80%) in growth turbidity compared to the control (antifungal agent-free) at 620 nm.

### Fungal Growth Curve

*C. albicans* SC5314 was cultured in RPMI 1640 medium containing 11g (0, 0.5, and 1 × 10^–3^ mg/mL) with an inoculums size of 10^6^ cells/mL. Cells were cultured at 30°C with constant shaking and optical density at 600 nm were measured after incubating for 0, 1, 2, 4, 8, 12, 16, 24, and 32 h (Kesavan et al., 2005). Three independent experiments were performed.

### Viability Curve

*C. albicans* SC5314 was cultured in RPMI 1640 medium containing 11g (0.0625 × 10^–3^, 0.125 × 10^–3^, or 0.25 × 10^–3^ mg/mL) with an inoculum size of 1 × 10^5^ cells/mL. Cells were cultured at 30°C with constant shaking. After 0, 12, 24, 36, and 48 h of incubation, a fraction was obtained from each solution and plated on a Sabouraud dextrose agar (SDA) plate. The numbers of colonies were counted after incubating at 30°C for 48 h. Three independent experiments were performed (Quan et al., 2006).

### Filamentation

*C. albicans* SC5314 was incubated in Sabouraud dextrose broth (SDB) at 25°C for 3-day to obtain a saturated culture. After washing three times with PBS, the cells were then resuspended in RPMI 1640 medium with 11g (0.25 × 10^–3^ or 0.5 × 10^–3^ mg/mL) to a concentration of 5 × 10^5^ cells/mL. The cells were pictured after further incubating at 37°C for 3 h.

### Staining and Confocal Microscope

Overnight cultures of *C. albicans* SC5314 were incubated in YPD medium with or without 11g at 30°C, favoring yeast-form growth. For hyphal growth, *C. albicans* SC5314 (1 × 10^6^ cells/mL) was incubated in Spider medium with or without 11g at 37°C for 3 h. Cells were then washed three times with PBS and then stained with Concanavalin A (ConA; Life Technologies, Carlsbad, CA, United States) to visualize mannan, anti-β-glucan primary antibody (Biosupplies, Bundoora, Australia) followed by Cy3-labeled secondary antibody (Life Technologies) to visualize β-(1, 3)-glucan and CFW (Life Technologies) to visualize chitin (Wheeler and Fink, 2006; Victoria et al., 2010). Microscopy was performed on a Leica confocal microscope (Wetzlar, Gernamy) with a 60 × oil objective.

### Cell Surface Hydrophobicity

Overnight cultures of *C. albicans* SC5314 were incubated in YPD medium with or without 11g (0.0313 × 10^–3^ mg/mL) at 30°C. Then, *C. albicans* cells were washed three times with PBS and resuspended in PBS (OD_600_ = 1.0). Then, cyclohexane (0.75 mL) was added to *C. albicans* cell suspension (3 mL). After vortex mixing for 3 min, the *C. albicans* cell suspension was settled at room temperature for 20 min. OD_600_ of the aqueous phase was measured by NANODROP2000 Ultraviolet spectrophotometer (Thermo Fisher Scientific, Waltham, MA, United States). Relative cell surface hydrophobicity (CSH) = (OD_600_ of the control – OD_600_ after octane overlay)/OD_600_ of the control × 100% (Zhang et al., 2016).

### Primary Murine Peritoneal Macrophage Preparation

Primary murine peritoneal macrophages were prepared as described previously (Saijo et al., 2007). Briefly, mice were injected intraperitoneally with 2 mL thioglycollate (3%; Merck, Darmstadt, Germany). Three days later, peritoneal cells were collected by washing with PBS containing 0.5 mM EDTA (BioDee, Beijing, China) and then cultured in RPMI 1640 medium containing 10% fetal bovine serum (FBS; Thermo Scientific, Waltham, MA, United States). After 2-day cultivation, adherent cells were used as primary murine peritoneal macrophages.

### Phagocytosis Rate and Phagocytosis Index Tests

In this paper, we investigated the phagocytosis rate and phagocytosis index by Wright-Giemsa staining tests. Here, we chose primary murine peritoneal macrophages as effector cells, *C. albicans* SC5314 and 11g (0.0313 × 10^–3^ mg/mL)-treated *C. albicans* SC5314 as target cells. Macrophages were cultured in 48-well plates at 37°C for 48 h. Overnight cultures of *C. albicans* SC5314 and 11g (0.0313 × 10^–3^ mg/mL)-treated *C. albicans* SC5314 were harvested and washed three times with PBS. After discarding the culture medium of macrophages, *C. albicans* SC5314 or 11g (0.0313 × 10^–3^ mg/mL)-treated *C. albicans* SC5314 was added to macrophages [multiplicity of infection (MOI) = 30] and cultured at 37°C for 0.5 h. After discarding the culture medium, 48-well plates were washed by PBS for two times. Cells were stained by rapid Wright-Giemsa staining solution (BBI Life Sciences Corporation, Shanghai, China). Then cells were washed by PBS for three times. Cells were photographed and counted by a microscope (Nicola and Casadevall, 2012). Phagocytosis rate = (number of macrophages phagocytizing fungi in 200 macrophages/200) × 100%; Phagocytosis index = number of fungi phagocytized by 200 macrophages/200.

### Fungi Killing Efficiency Tests

Firstly, primary murine peritoneal macrophages were incubated in 24-well plates at 37°C for 48 h. Overnight cultures of *C. albicans* SC5314 and 11g (0.0313 × 10^–3^ mg/mL)-treated *C. albicans* SC5314 were harvested and washed three times with PBS. After discarding the culture medium of macrophages, *C. albicans* SC5314 or 11g (0.0313 × 10^–3^ mg/mL)-treated *C. albicans* SC5314 (MOI = 1) were added to 24-well plates and incubated at 37°C for 6 h. Then, fungi and macrophages suspensions from each well were distributed on the surface of SDA plates and incubated at 30°C for 48 h. Then the CFU of *C. albicans* SC5314 on each SDA plate was counted. We recorded the group of *C. albicans* SC5314 as group A, the group of 11g-treated *C. albicans* SC5314 as group B, the group of *C. albicans* SC5314 + macrophages as group C, and the group of 11g-treated *C. albicans* SC5314 + macrophages as group D. The killing efficiency of macrophages to *C. albicans* SC5314 = (A – C)/A × 100%. The killing efficiency of macrophages to 11g-treated *C. albicans* SC5314 = (B – D)/B × 100%. Three independent experiments were performed.

### Western Blotting

Overnight cultures of *C. albicans* SC5314 incubated in YPD medium with or without 11g were harvested and washed three times with PBS. The fungi were then exposed to five doses of 100 mjoules/cm^2^ ultraviolet (UV) radiation in a CL-1000 UV-crosslinker (UVP; Upland, United States) (Victoria et al., 2010). After UV-inactivation, fungi were washed extensively, renormalized by hemocytometry, added to cultures of the primary murine peritoneal macrophages preincubated with or without laminarin (Sigma), and then incubated at 37°C for the indicated time. MOI = 5. Primary murine peritoneal macrophages were harvested, stimulated, and lysed in lysis buffer for total cell lysates. In nuclear extracts immunoblotting experiments, cells were lysed in lysis buffer and extracted by extraction buffer. Both the total cell lysates and nuclear extracts were subjected to SDS-PAGE, transferred to nitrocellulose membrane, and immunoblotted with the indicated antibodies (Gorjestani et al., 2012).

### Cytokine Detection

After macrophage and UV-inactivated fungus interaction, supernatants of primary murine peritoneal macrophages were harvested, and cytokines concentrations were measured by enzyme-linked immunosorbent assay (ELISA) according to manufacturer’s instructions (eBioscience, San Diego, CA, United States). Three independent experiments were performed.

### Cytotoxicity Assay

For cytotoxicity assay, rat cardiac myocytes (H9C2; 8 × 10^3^ cells/well) were cultured in 96-well plates and incubated in DMEM (HyClone, Logan City, United States), supplemented with 10% FBS at 37°C in the presence of 5% CO_2_. The following day, a CCK-8 assay was performed, as described previously (Huang et al., 2018). Briefly, H9C2 were incubated in DMEM with or without 11g at 37°C for 24 h. After discarding medium with 11g, washing cells two times with PBS, and adding fresh medium with CCK-8 solution, cells were further incubated at 37°C for 1 h. Absorbance at 450 nm was measured by ELISA Plate Reader (Thermo Scientific). Three independent experiments were performed.

### Murine Systemic Candidiasis Model and Treatment

*C. albicans* SC5314 was cultured in YPD medium at 30°C for 12 h. After washing them three times with PBS, cells were resuspended in physiological saline. Infection was induced by injecting a *C. albicans* suspension into the lateral tail vein (5 × 10^5^ CFU/mouse). 11g (4 mg/kg) or vehicle was administered intraperitoneally 2 h later and was injected once a day for 5 consecutive days (Hata et al., 2011). Survival rates were monitored for 35 days following infection. For measuring fungal burden, serial dilutions of paired kidneys and livers were plated on SDA plates on Day 5 after infection, and further incubated at 30°C for 48 h. For histopathological analysis, paired kidneys were fixed in 10% neutral buffered formalin on Day 5 after infection, and then stained with hematoxylin and eosin (H&E) and periodic acid-Schiff (PAS) to reveal inflammatory cell infiltration and fungal hyphae. Three independent experiments were performed.

### Statistical Analysis

Three biological replicates were performed for all the experiments in this paper, unless otherwise indicated. Statistical analyses were performed using the Prism software, version 5.01 (GraphPad software, San Diego, CA, United States). Log-rank test was used to analyze the difference between the survival curves. The Mann-Whitney U test was used to analyze the difference between the two groups. Two-way ANOVA or one-way ANOVA was used to analyze the difference between multiple groups. A quantitative analysis of mean fluorescent intensity was performed using Image J (Bethesda, MD, United States). A densitometric analysis of Western blot images was performed using Image J. Statistical significance was set at probability (*P*) value of less than 0.05, 0.01, or 0.001, indicated by ^∗^, ^∗∗^, or ^∗∗∗^, respectively.

## Results

### MIC_80_ of 11g *in vitro*

**FIGURE 1:**
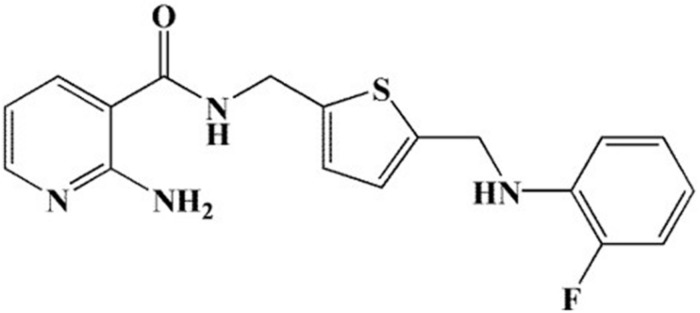
Chemical structure of 11g.

In 2017, our group designed and synthesized a series of novel 2-aminonicotinamide derivatives and reported that compound 11g (Figure 1) is a promising new antifungal agent with potent and broad-spectrum antifungal activity *in vitro* (Ni et al., 2017). Here, in order to expand the antifungal spectrum of 11g, we investigated the antifungal activity of 11g against 17 clinical isolates of *Candida* species.

Table 1 shows the MIC_80_ of 11g against 4 clinical isolates of Fluconazole-susceptible *Candida albicans*, 8 clinical isolates of Fluconazole-resistant *Candida albicans*, 2 clinical isolates of *Candida krusei*, 2 clinical isolates of *Candida glabrata*, and 1 clinical isolates of *Candida tropicalis in vitro*. 11g showed potent antifungal activity not only against fluconazole-susceptible *C. albicans* (MIC_80_ of 11g ≤ 0.5 × 10^–3^ mg/mL), but also against fluconazole-resistant *C. albicans* (MIC_80_ of 11g ≤ 0.5 × 10^–3^ mg/mL). In addition, data show that 11g exhibited strong antifungal effect against all the strains tested. Moreover, it should be noted that most of the strains tested here were much more sensitive to 11g than fluconazole. These data further prove that 2-aminonicotinamide derivatives (11g) have potent and broad-spectrum antifungal activity *in vitro*.

**TABLE 1:** *In vitro* antifungal activities of 11g against *Candida albicans*, *Candida krusei*, *Candida glabrata*, and *Candida tropicalis*.

Strain	MIC_80_ (×10^–3^ mg/mL)	
	
	11g	Fluconazole
**Fluconazole-susceptible *Candida albicans***		
*Candida albicans* SC5314	0.0313	0.5
*Candida albicans* UCA32	0.25	0.5
Candida albicans 26^#^	0.5	0.5
*Candida albicans* 9^#^	0.5	0.25
**Fluconazole-resistant *Candida albicans***		
*Candida albicans* 385	0.125	>64
*Candida albicans* UCA42	0.125	>64
*Candida albicans* 0606341	0.125	>64
*Candida albicans* UCA45	0.25	>64
*Candida albicans* UCA4	0.125	64
*Candida albicans* UCA3	0.25	64
*Candida albicans* 0512644	0.5	64
*Candida albicans* UCA1	0.125	64
***Candida krusei***		
*Candida krusei* ATCC2340	2	64
*Candida krusei* ATCC2159	1	16
*Candida glabrata*		
*Candida glabrata* ATCC1182	0.5	4
*Candida glabrata* ATCC28226	0.5	4
***Candida tropicalis***		
*Candida tropicalis* 2718	0.5	>64

*^#^are two names of clinical isolates Fluconazole-susceptible *Candida albicans*.*

### Effect of 11g on *C. albicans* Growth and Virulence

**FIGURE 2:**
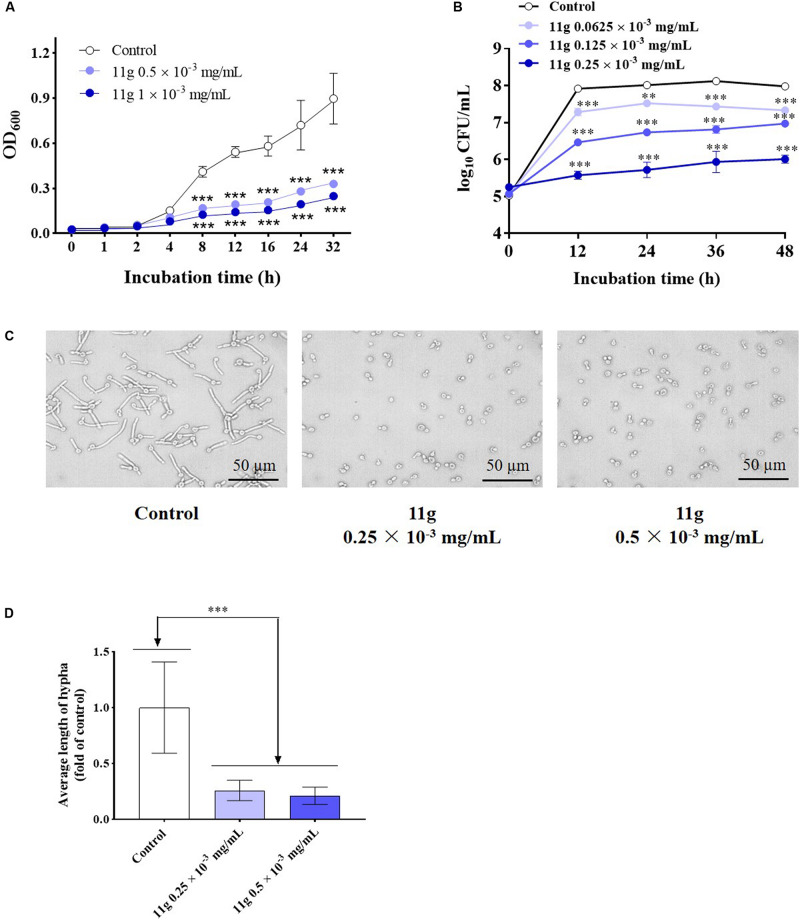
The effect of 11g on the growth and invasive ability of *C. albicans in vitro*. **(A)** Fungal growth curves of *C. albicans* SC5314 incubated with 11g (0, 0.5, and 1 × 10^–3^ mg/mL; *n* = 3; mean ± SD; two-way ANOVA with Dunnett test; ***P < 0.001). **(B)** Viability curves of *C. albicans* SC5314 incubated with 11g (0.0625 × 10^–3^, 0.125 × 10^–3^, and 0.25 × 10^–3^ mg/mL; *n* = 3; mean ± SD; two-way ANOVA with Dunnett test; **P < 0.01; ^∗∗∗^*P* < 0.001). **(C)** Microscopic images of *C. albicans* SC5314 incubated with 11g (0.25 × 10^–3^ and 0.5 × 10^–3^ mg/mL) at 37°C for 3 h. **(D)** A quantitative analysis of hypha length was measured by Image J (*n* = 50; mean ± SD; one-way ANOVA with Dunnett test; ^∗∗∗^*P* < 0.001).

Next, we investigated the effect of 11g on the growth and virulence of *C. albicans*. Fungal growth curves show that 11g at 0.5 × 10^–3^ and 1 × 10^–3^ mg/mL could potently inhibit the growth of *C. albicans* during 32 h culture. In addition, 11g-incubated *C. albicans* growth in a concentration-dependent manner (Figure 2A). Data show that the doubling time of control group was 6.2 h. However, the doubling times of 11g (0.5 and 1 × 10^–3^ mg/mL)-treated groups were 8.7 and 9.8 h, respectively, which was significantly longer than that of control group. Results above show that 11g could significantly inhibit the growth of *C. albicans* SC5314 *in vitro*. Viability curves show that 11g at 0.0625 × 10^–3^, 0.125 × 10^–3^, and 0.25 × 10^–3^ mg/mL could potently inhibit the viability of *C. albicans* after 48 h culture. Additionally, 11g inhibited the viability of *C. albicans* in a concentration-dependent manner (Figure 2B). We further examined the effect of 11g on fungi virulence. It has been reported that the hypha is a virulence factor, which plays an important role in the invasion and infection of *C. albicans* (Kumamoto and Vinces, 2005). Figures 2C,D shows that 11g (0.25 × 10^–3^ mg/mL) could significantly shorten the length of the hypha. All these data above suggest that 11g exhibits potent inhibition effect on growth and virulence of *C. abicans*.

In April 2019, our research group published an article named “Study on the mechanism of NT-89 against *Candida albicans* based on quantitative proteomics (in Chinese)” (Liu et al., 2019). Here, NT-89 was the former name of 11g. In this study, we firstly extracted the total protein of *C. albicans* SC5314 and 11g-treated *C. albicans* SC5314. Then, we discussed the effect of 11g on the protein expression of *C. albicans* SC5314 by iTRAQ tests. We analyzed the top 20 proteins with reduced relative expression in 11g-treated *C. albicans* SC5314. Data show that there were 5 GPI-anchored proteins among these 20 proteins, which was consistent with our conjecture that 11g could inhibit the synthesis of GPI (Ni et al., 2017). Among the above 20 proteins, Ywp1p and Pga10p decreased the most. Ywp1p plays a key role in the dispersal in host, adhesion, and biofilm formation (Granger et al., 2005). Pga10p plays a key role in the biofilm formation (Klis et al., 2009). Furthermore, in 11g-treated *C. albicans* SC5314, the relative expression of some proteins like Hsp family proteins and adhesin-like proteins were also significantly decreased (Liu et al., 2019). Data above indicated that the virulence of 11g-treated *C. albicans* SC5314 was decreased, which was consistent with the results of hypha formation experiments.

### Effect of 11g on *C. albicans* Cell Wall Polysaccharides

Previous studies have shown that *C. albicans* cell wall consists of two layers: The outer layer is enriched with mannoprotein, and the inner layer contains the skeletal polysaccharides β-(1, 3)-glucan and chitin (Gow et al., 2011). In 2017, our group investigated the ultrastructure of fungal cell wall by transmission electron microscopy (Ni et al., 2017). We reported that the dense mannoprotein coats became thinner, and GPI content reduced after being incubated with 11g (Ni et al., 2017).

**FIGURE 3:**
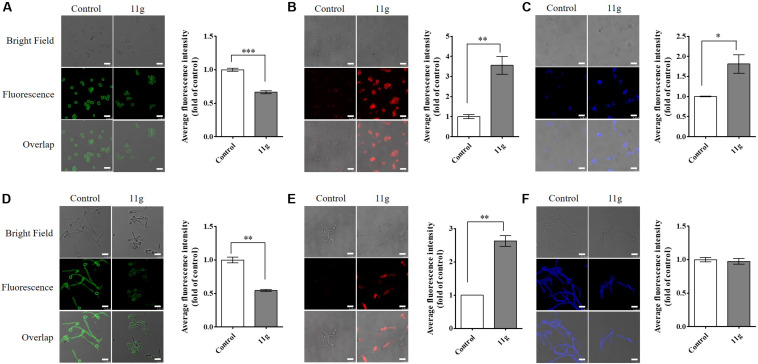
The effect of 11g on cell wall polysaccharides. *C. albicans* SC5314 was grown in YPD medium **(A–C)** overnight, favoring yeast-form growth or spider medium **(D–F)** for 3 h, favoring hyphal growth. Then cultures grown with or without 11g (0.0313 × 10^–3^ mg/mL) were stained with ConA to visualize mannan (green, A and D), anti-β-glucan primary antibody followed by Cy3-labeled secondary antibody to visualize β-1, 3-glucan (red, B and E), or CFW to visualize chitin (blue, **C,F**) by a confocal laser-scanning microscope. Scale bars, 10 μm. A quantitative analysis of mean fluorescent intensity was measured by Image J (*n* = 3; mean ± SD; Mann-Whitney *U* test; ^∗^*P* < 0.05; ^∗∗^*P* < 0.01; ^∗∗∗^*P* < 0.001).

Here, in order to further investigate the effect of 11g on fungal cell wall polysaccharides, we stained fungi with fluorescent dyes and then observed cell wall polysaccharides by confocal laser-scanning microscopy. In this paper, we stained fungi with ConA to visualize mannan and anti-β-glucan primary antibody followed by Cy3-labeled secondary antibody to visualize β-(1, 3)-glucan. In 11g-treated yeast-form *C. albicans*, the fluorescence intensity of ConA is weakened, suggesting mannan content is decreased (Figure 3A); the fluorescence intensity of Cy3 is enhanced, suggesting β-(1, 3)-glucan is exposed (Figure 3B). Consistent with yeast-form cells, the same changes occurred to hyphal mannoprotein coat (Figure 3D) and β-glucan layer (Figure 3E). In addition, we stained fungi with CFW to visualize chitin. CFW could specifically combine with chitin and emitted blue fluorescence at a 355-nm excitation wavelength. In 11g-treated yeast-form *C. albicans*, the fluorescence intensity of CFW is enhanced, suggesting chitin content has a compensatory increase (Figure 3C). However, there was no significant differences in fluorescence intensity of CFW between 11g-treated hypha and control group (Figure 3F). What’s more, 11g also shortened the length of filaments, which is consistent with the previous results in this paper (Figures 3D–F). Together, these data indicate that 11g can remodel the architecture of fungal cell wall, which is consistent with our previous study (Ni et al., 2017). In addition, we also investigated the effect of 11g on cell surface hydrophobicity. Data show that cell surface hydrophobicity was significantly increased in 11g (0.0313 × 10^–3^ mg/mL)-treated *C. albicans* SC5314 (Supplementary Figure S2). Data above indicate that 11g affected the surface property of *C. albicans* such as hydrophobicity, which confirms the conclusion that 11g led to the remodeling of fungi cell wall.

The small molecule compound 11g reported in this paper is derived from the structure optimization of the published GPI biosynthesis inhibitors BIQ, G884, 10b, and E1210. It has been reported that all of these four compounds (BIQ, G884, 10b, and E1210) could inhibit the function of Gwt1p, the key enzyme in GPI synthesis pathway in fungi, and finally exhibit antifungal activity (Tsukahara et al., 2003; Nakamoto et al., 2010; Hata et al., 2011; Mann et al., 2015; Zhao et al., 2018, 2019). Therefore, we inferred that Gwt1p might be the target of 11g. In order to verify our conjecture, we constructed *GWT1* heterozygous mutant *C. albicans* strain (*GWT1/gwt1△*). Results of spot assay show that *C. albicans* with *GWT1/gwt1△* exhibited higher sensitivity to 11g, indicating that Gwt1p might be the target of 11g (data not shown). In the future experiments, the target of 11g will be further verified by *GWT1* gene overexpression experiments and gene screening experiments. The research on 11g antifungal target is another story to be reported in later details from our group.

### 11g Enhances *in vitro* Innate Immune Responses to *C. albicans*

Previous studies suggest that β-(1, 3)-glucan is a predominant pathogen-associated molecular patterns (PAMP) in *C. albicans* cell wall, which is usually masked by the mannoprotein coats (Poulain and Jouault, 2004; Gow et al., 2011). As we found out that 11g can unmask the β-(1, 3)-glucan, we reasoned that 11g can enhance the immunogenicity of *C. albicans*, leading to the boost of innate immune cell responses (Brown et al., 2003; Gantner et al., 2003; Netea et al., 2008).

To validate our hypothesis, we first investigated the phagocytosis rate and phagocytosis index by Wright-Giemsa staining tests. Here, we chose primary murine peritoneal macrophages as effector cells. *C. albicans* SC5314 and 11g (0.0313 × 10^–3^ mg/mL)-treated *C. albicans* SC5314 as target cells. Data show that there was no significant difference in phagocytosis rate between the two groups. The phagocytosis rate was 100% in both of these two groups, indicating that all macrophages in these two groups exhibited phagocytic behavior. However, the phagocytosis index of macrophages to 11g-treated *C. albicans* SC5314 was significantly higher than that of *C. albicans* SC5314, indicating that 11g enhanced the phagocytic function of macrophages to fungi (Supplementary Figure S3). In addition, we investigated the killing efficiency of macrophages to *C. albicans* SC5314. Data show that the average killing efficiency of macrophages to *C. albicans* SC5314 and 11g-treated *C. albicans* SC5314 is 21.2% and 39.9%, respectively. The average killing efficiency of macrophages to 11g-treated *C. albicans* SC5314 is significantly higher than that of macrophages to *C. albicans* SC5314, which is consistent with the result of phagocytosis index (Supplementary Figure S4).

**FIGURE 4:**
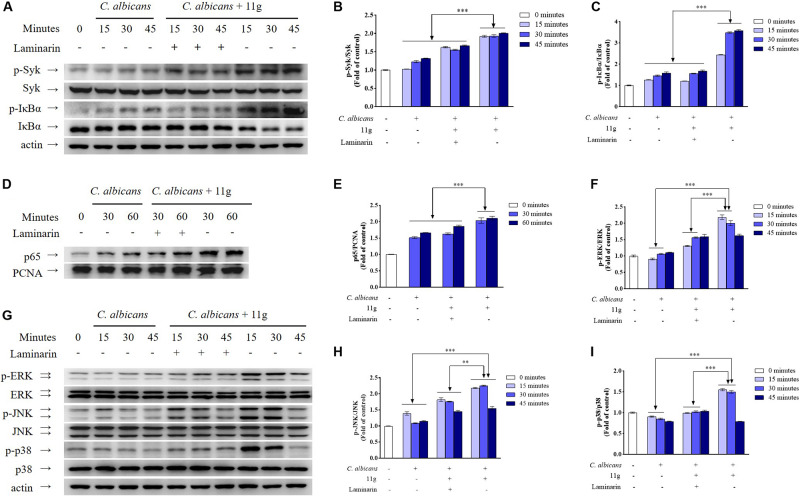
11g-treated *C. albicans-*induced NF-κB and MAPK activation in innate immune cells. Primary murine peritoneal macrophages were incubated with or without laminarin (500 × 10^–3^ mg/mL) at 37°C for 30 min. The macrophages were then stimulated with 11g (0.0313 × 10^–3^ mg/mL) or vehicle-treated *C. albicans* (MOI = 5) for the indicated times. The total cell lysates **(A,F)** and nuclear extracts **(D)** were analyzed by immunoblotting using the indicated antibodies. A densitometric analysis (**B,C** for **A**; **E** for **D**; **G–I** for **F**) was measured by Image J (*n* = 3; mean ± SD; two-way ANOVA with Tukey test; ^∗∗^*P* < 0.01; ^∗∗∗^*P* < 0.001).

Then, we investigated the effect of 11g-treated *C. albicans* on signaling pathways activation in innate immune cells. Results show that 11g-treated *C. albicans* can enhance the phosphorylation of Syk and IκBα, as well as the degradation of IκBα in macrophages (Figures 4A–C). Moreover, 11g-treated *C. albicans* can also promote the nuclear translocation of p65 subunit in macrophages (Figures 4D,E). What’s more, these activations can be significantly inhibited by laminarin, a specific inhibitor of Dectin-1 (Figures 4A–E; Gantner et al., 2005). These results indicate that 11g-treated *C. albicans* can enhance Dectin-1-dependent NF-κB-signaling activation in macrophages. In this study, we also studied the effect of 11g-treated *C. albicans* on MAPK-signaling activation in macrophages. Figures 4F–I shows that 11g-treated *C. albicans* can enhance the phosphorylation of ERK, JNK, and p38 in macrophages. Consistently, these activations can also be significantly inhibited by laminarin. These data suggest that 11g-treated *C. albicans* can enhance Dectin-1-dependent MAPK-signaling activation in macrophages.

**FIGURE 5:**
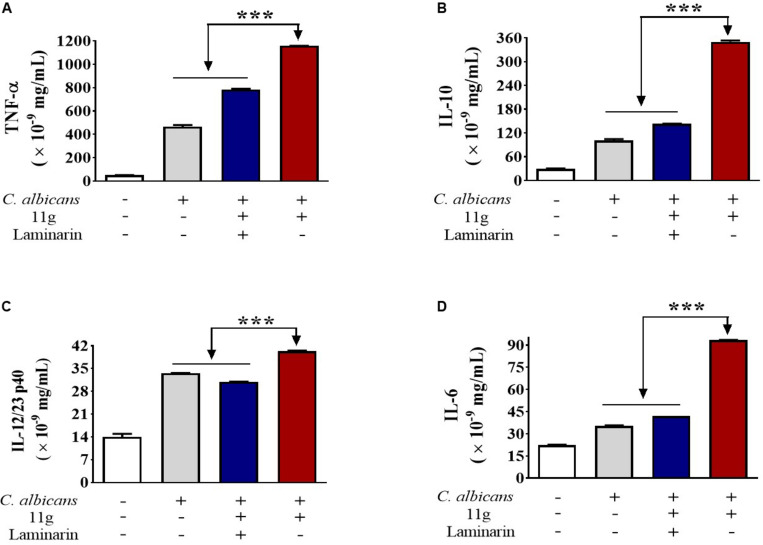
11g-treated *C. albicans* induced cytokines secretion in innate immune cells. Primary murine peritoneal macrophages were incubated with or without laminarin (500 × 10^–3^ mg/mL) at 4 °C for 30 min. The macrophages were then stimulated with 11g (0.0313 × 10^–3^ mg/mL) or vehicle-treated *C. albicans* (MOI = 5) for 6 h. Concentrations of TNF-α **(A)**, IL-10 **(B)**, IL-12/23 p40 **(C)**, and IL-6 **(D)** in supernatant were measured by ELISA (*n* = 3; mean ± SD; one-way ANOVA with Bonferroni post-test; ***P < 0.001).

Next, we investigated the effect of 11g-treated *C. albicans* on cytokines secretion in macrophages. We found that 11g-treated *C. albicans* could increase the secretion of TNF-α (Figure 5A), IL-10 (Figure 5B), IL-12/23 p40 (Figure 5C), and IL-6 (Figure 5D) in macrophages after 6 h post-infection. In addition, these activations were significantly inhibited by laminarin. These data suggest that 11g-treated *C. albicans* can elicit Dectin-1-dependent cytokines secretion in macrophages.

### Cytotoxicity of 11g *in vitro*

**FIGURE 6:**
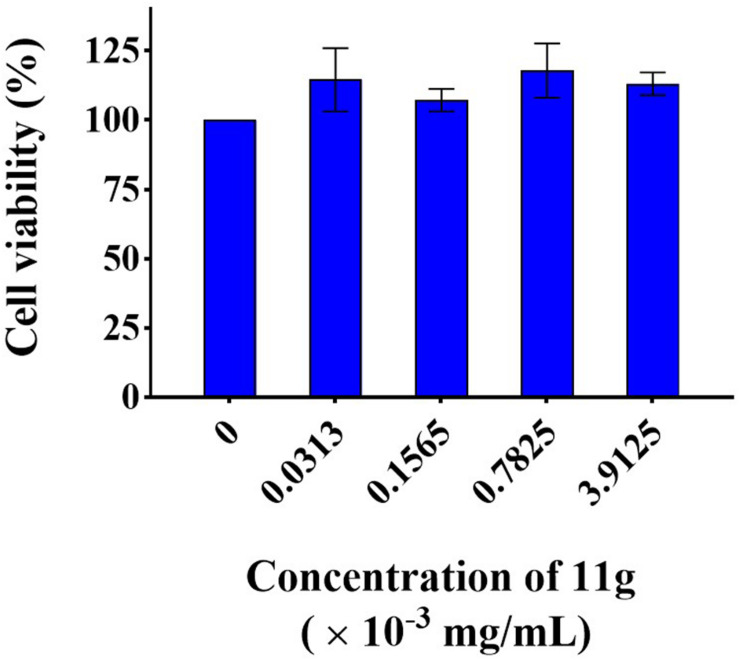
Cytotoxicity of 11g to H9C2. Cells were treated with 11g for 24 h, after which relative viable cell number was measured by CCK-8 assay (*n* = 3; mean ± SD; one-way ANOVA with Bonferroni post-test).

In order to infer the safety of 11g, we investigated the mammalian cytotoxicity of 11g *in vitro* (Figure 6). The concentrations of 11g incubated with H9C2 were at 1×, 5×, 25×, and 125 × MIC_80_ (MIC_80_ = 0.0313 × 10^–3^ mg/mL), respectively. Results show that 11g exhibited no significant effect on cell viability of H9C2 at all the concentrations tested, which means the cytotoxicity of 11g is low *in vitro*.

### Protective Effect of 11g Against Fungal Infection *in vivo*

**FIGURE 7:**
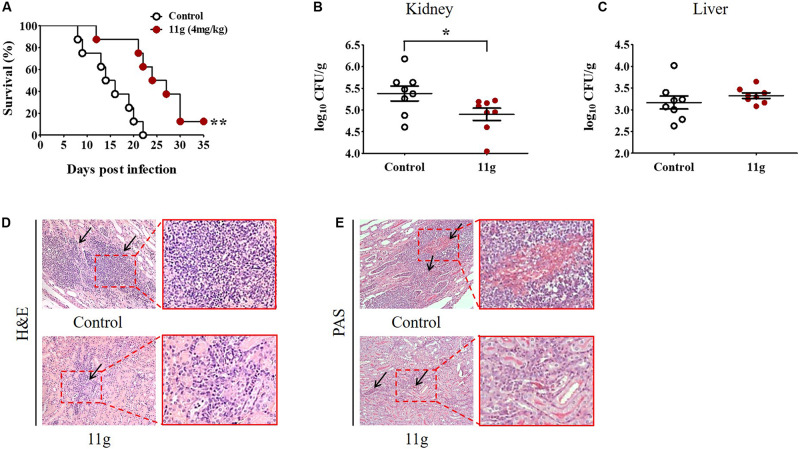
11g exerts protective effects in mice with systemic candidiasis. Mice (n = 8 per group) were intravenously infected with 5 × 10^5^ CFU of *C. albicans* SC5314. Then 11g (4 mg/kg) was administered intraperitoneally 2 h later. Antifungal agents were injected once a day for 5 consecutive days. **(A)** Survival rates were monitored for 35 days after infection (*n* = 8; log-rank test; **P < 0.01). **(B,C)** Fungal burdens of paired kidneys **(B)** and livers **(C)** were measured on day 5 post-infection. Fungal burden was determined as log_10_ CFU/g (*n* = 8; mean ± SD; Mann-Whitney *U* test; *P < 0.05). **(D,E)** Histopathological analysis of kidneys from 11g treated group. **(D)** H&E-stained sections. **(E)** PAS-stained sections. Black arrows in **(D)** indicate inflammatory cell infiltration and in **(E)** indicate hyphae. Magnification = 200×. Data are representative of three independent experiments.

To investigate the protective effect of 11g *in vivo*, mice were intravenously infected with *C. albicans* to prepare systemic candidiasis, and then treated with 11g (4 mg/kg) for 5 consecutive days. Results of survival rates show that mice treated with vehicle all died within 22 days post-infection. The median survival of mice in control group was attained at 14 days and at 24 days in 11g group (Figure 7A). These results suggest that 11g exhibited significantly protective effect against systemic candidiasis. Data of fungal burden of paired kidneys show that the median value of fungal burden is significantly lower in 11g group than in control group (Figure 7B). At the same time, we tested fungal burden in livers either. However, there was no significant differences in fungal burden between 11g group and control group (Figure 7C). Histopathological analysis of kidneys shows that there were less inflammatory cells and fungal hyphae in 11g group than in control group (Figures 7D,E). These data suggest that 11g has potent protective effect against fungal infection *in vivo*.

In this paper, 11g (4 mg/kg) was used to treat mice with systemic candidiasis. 11g showed significant inhibition effect on fungal burden in kidneys, but not in livers. We inferred that this might be due to the low experimental concentration of 11g used in this paper. Therefore, we increased the experimental concentration of 11g in the follow-up experiment. Higher concentrations (8, 16, and 32 mg/kg) of 11g were used to treat mice with systemic candidiasis. Data show that the mice survival rate increased with the increase of the dose concentration. In the fourth week after infection, the survival rate of 11g (8 mg/kg) treatment group increased to 50%, and the survival rate of 11g (16 or 32 mg/kg) treatment group increased to 75% (data not shown). The fungal burden of different organs in 11g-treated mice (at higher concentrations, such as: 8, 16, and 32 mg/kg) with systemic candidiasis will be investigated in the future. A detailed report about the therapeutic effect of 11g (higher concentrations, such as: 8, 16, and 32 mg/kg) will be discussed in the next paper by our group.

## Discussion

In recently years, a series of GPI biosynthesis inhibitors have been reported by researchers (Tsukahara et al., 2003; Nakamoto et al., 2010; Miyazaki et al., 2011; Mann et al., 2015). In 2003, Tsukahara et al. reported a GPI-anchored mannoproteins inhibitor (BIQ), which targets *GWT1* gene product (Tsukahara et al., 2003). On the basis of optimizing BIQ, Eisai Co. Ltd reported two novel 2-aminopyridine derivatives (10b and E1210) in 2010 and 2011, which have been showed as effective *GWT1* protein inhibitors (Nakamoto et al., 2010; Miyazaki et al., 2011). Three years later, another GPI inhibitor agent named G884 was reported by Mann et al. (2015), which proved to have an effective inhibition effect on Gwt1-mediated acylation. In the course of further studies on the structure–activity relationship of these GPI biosynthesis inhibitors, our group designed and synthesized a series of 2-aminonicotinamide derivatives (Ni et al., 2017). Among them, 11g is the most promising antifungal agent, which arouses our interest in further research.

It has been reported that the *C. albicans* cell wall consists two layers (Gow et al., 2011). Skeletal polysaccharides β-(1, 3)-glucan and chitin are located in the inner layer and are covered by mannoprotein (outer layer) (Gow et al., 2011). Most CWPs are GPI-CWPs. Most often, they are covalently attached to the inner layer through a GPI remnant and β-(1, 6)-glucan polymers (Richard and Plaine, 2007; Chaffin, 2008). Therefore, GPI absence would remove the attachment sites of GPI-CWPs, leading to the destruction of mannoprotein layer and the exposure of β-glucan.

In the published paper “Study on the mechanism of NT-89 against *Candida albicans* based on quantitative proteomics (in Chinese),” we analyzed the effect of 11g on the protein expression of *C. albicans* SC5314 by iTRAQ tests (Liu et al., 2019). Data show that the expression of some GPI-anchored proteins (such as RHD3) were significantly decreased in 11g-treated *C. albicans* SC5314. RHD3 is a GPI-anchored cell wall protein in *C. albicans* SC5314. It has been reported that *rhd3* mutants display a significant reduction of cell wall mannan, which is consistent with the results of confocal experiments in this paper (de Boer et al., 2010). At the same time, the expression of some proteins (such as PMI1 and PMT1) was significantly increased in 11g-treated *C. albicans* SC5314. PMI1 is a phosphomannose isomerase, which is involved in biosynthesis of the cell wall (Smith et al., 1995); PMT1 is a protein mannosyltransferase responsible for the initiation of O-mannosylation, which plays an important role in maintaining the integrity of cell wall (Prill et al., 2005). Based on the above data, we inferred that 11g could affect the structure of mannose layer in cell wall by inhibiting the GPI biosynthesis pathway. At the same time, this effect triggered the feedback regulation in fungi, leading to the repair of mannose layer and the expression enhancement of some mannose proteins in cell wall. Therefore, 11g could result in the remodeling of fungi cell wall and the exposure of partial glucan. The interaction of mannose and glucan enhanced the immunogenicity of 11g-treated *C. albicans* SC5314.

It is known that β-glucan is a predominant PAMP in fungal cell wall. The innate immune system can clear fungi after recognizing β-glucan in host-pathogen interaction (Poulain and Jouault, 2004). However, the truth is that a fungus often escapes from the host immune recognition by camouflaging the majority of its β-glucan layer (Wheeler and Fink, 2006). Here, as an effective antifungal agent, 11g could enhance fungal immunogenicity by exposing the β-glucan layer. Results show that 11g-treated *C. albicans* activated the NF-κB- and MAPK-signaling cascades, and also elicited specific cytokine (TNF-α, IL-10, IL-12/23 p40, and IL-6) secretions from macrophages. Previous studies show that TNF-α or IL-12 could facilitate Th1 cells differentiation and activation; IL-6 or IL-23 could facilitate Th17 cells differentiation and activation (Aggarwal et al., 2003; Trinchieri, 2003). The activation of Th1 and Th17 cells would trigger inflammatory responses, leading to the recruitment of humoral and cellular immune factors (LeibundGut-Landmann et al., 2012; Cassone, 2013). IL-10 could facilitate CD^4+^CD^25+^T_reg_ cell proliferation, and trigger immunosuppression to modulate immune responses (Netea et al., 2004; Sutmuller et al., 2006). Additionally, our results also show that all the immune recognition activities induced by 11g were significantly inhibited by laminarin, suggesting these immune recognition activities were mainly Dectin-1-dependent, which is consistent with previous reports (Brown et al., 2002; Gantner et al., 2005; LeibundGut-Landmann et al., 2012).

It has been reported that laminarin is a specific inhibitor of Dectin-1(Gantner et al., 2005). Compared with the data of *C. albicans* SC5314 + 11g group, the activation of NF-κB- and MAPK-signaling pathway was significantly inhibited in *C. albicans* SC5314 + 11g + laminarin group. At the same time, the release of inflammatory cytokines (TNF-α, IL-10, IL-12/23 p40, and IL-6) was significantly decreased in *C. albicans* SC5314 + 11g + laminarin group, too. These data suggest that 11g-treated *C. albicans* SC5314 could activate Dectin-1-dependent NF-κB- and MAPK-signaling pathway and further elicit Dectin-1-dependent cytokines secretion in macrophages. However, compared with *C. albicans* SC5314 group, the release of inflammatory factors partially increased in *C. albicans* SC5314 + 11g + laminarin group. These data suggest that there existed some other signaling pathways resulting in the release of inflammatory factors in *C. albicans* SC5314 + 11g + laminarin group. Therefore, Dectin-1-dependent signaling pathway is one of the main signaling pathways activated by 11g-treated *C. albicans* in macrophages. Also, it should be noted that some mannan (a PAMP) still remained on the surface of fungi after 11g treatment. Therefore, we cannot exclude the possibility that other pattern-recognition receptors (PRRs) participated in the immune recognition process.

Mannan is a PAMP. It seems to directly contradict with the viewpoint that fungi reduce its immunogenicity by covering mannan on β-glucan layer. A possible explanation is that mannan could prevent the recognition of Dectin-1 to β-glucan, as well as the interaction between mannan-TLR and β-glucan-Dectin-1 pathways. It has been shown that mannan-TLR and β-glucan-Dectin-1 pathways exhibit potent synergistic effect on immune response. Therefore, although mannan could be recognized by immune cells, it cannot cause the very efficient activation of host immune system by itself (Netea et al., 2008).

In summary, our study shows that 11g is a potent antifungal agent both *in vitro* and *in vivo*. Further study shows that 11g could enhance the immunogenicity of fungi by unmasking β-glucan layer, activating the Dectin-1-dependent protective immune responses. Thus, 11g is a very promising antifungal drug, which is expected to be a candidate to expand the toolbox of fungal infection treatment. Further studies are expected to investigate the molecular target of 11g on GPI synthesis or transport process. In addition, Dectin-1 knockout mice will be used to further verify the mechanism of Dectin-1-dependent protective immune responses induced by 11g-treated *C. albicans*.

## Data Availability Statement

All datasets generated for this study are included in the article/Supplementary Material.

## Ethics Statement

The animal study was reviewed and approved by Institutional Animal Use and Care Committee of Tongji University.

## Author Contributions

All authors contributed toward data analysis, drafting and revising the manuscript, and agree to be accountable for all aspects of the work.

## Conflict of Interest

The authors declare that the research was conducted in the absence of any commercial or financial relationships that could be construed as a potential conflict of interest.

## Abbreviations

ConA: concanavalin A
CSH: cell surface hydrophobicity
CWPs: cell wall proteins
DMSO: dimethyl sulfoxide
ELISA: enzyme-linked immunosorbent assay
FBS: fetal bovine serum
GPI: glycosylphosphatidylinositol
GPI-CWPs: GPI-anchored cell wall proteins
H&E: hematoxylin and eosin
MIC: minimum inhibitory concentration
MOI: multiplicity of infection
PAMP: pathogen-associated molecular patterns
PAS: periodic acid-schiff
PRRs: pattern-recognition receptors
SDA: sabouraud dextrose agar
SDB: sabouraud dextrose broth
UV: ultraviolet
YPD: yeast peptone dextrose.
