# Supplementary material for: 11g, a Potent Antifungal Candidate, Enhances Candida albicans Immunogenicity by Unmasking β-Glucan in Fungal Cell Wall
Source: Front Microbiol. 2020 Jun 30;11:1324. doi: 10.3389/fmicb.2020.01324 (PMC7338940; doi:10.3389/fmicb.2020.01324)
Supplement: Supplementary file 1 [file Data_Sheet_1.pdf]

Supplementary materials

1 Supplementary Figures

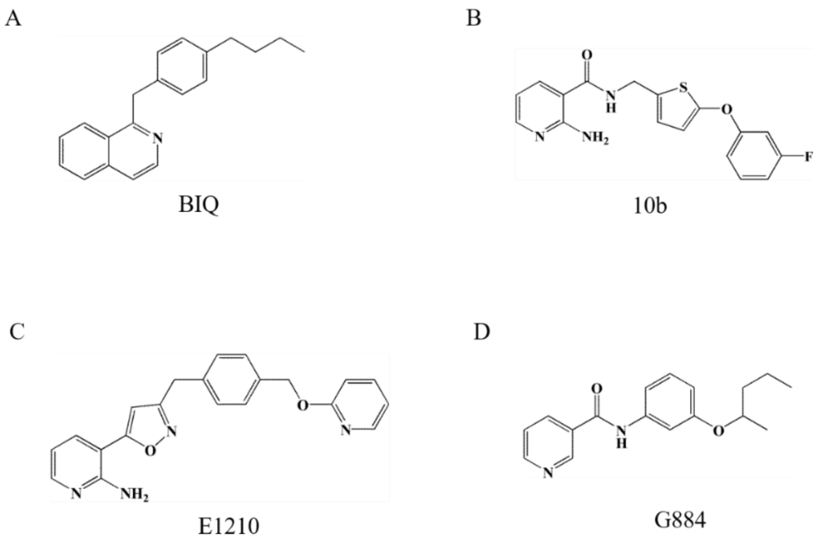

Supplementary Figure 1. Chemical structure of BIQ (A), 10b (B), E1210 (C), and G884 (D).

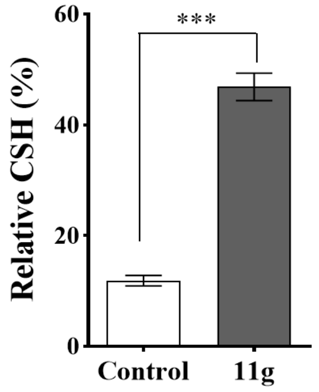

Supplementary Figure 2. Cell surface hydrophobicity in *C. albicans* SC5314 and 11g (0.0313 $\times 10^{-3}$ mg/mL)-treated *C. albicans* SC5314 (n = 3; Mean ±SD; Mann-Whitney U test; ***, $P < 0.001$).

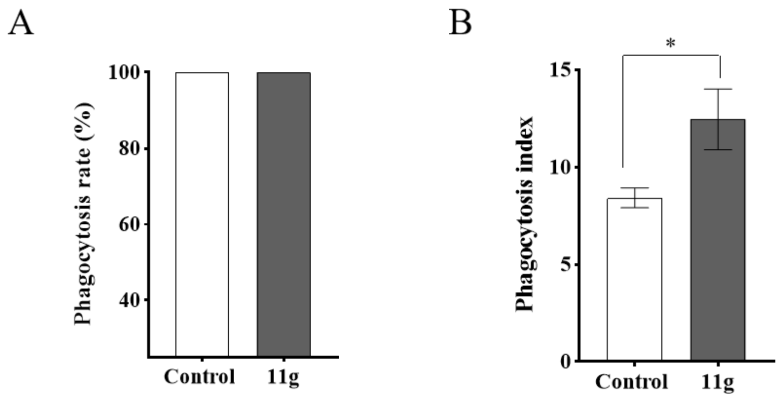

Supplementary Figure 3. The phagocytosis rate (A) and phagocytosis index (B) of macrophages to *C. albicans* SC5314 and 11g (0.0313 $\times 10^{-3}$ mg/mL)-treated *C. albicans* SC5314 (n = 3; Mean ±SD; Mann-Whitney U test; *, $P < 0.05$).

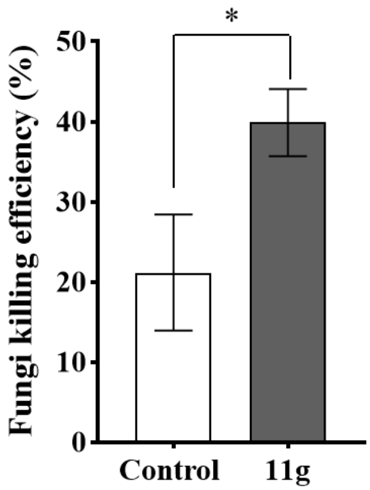

Supplementary Figure 4. The killing efficiency of macrophages to *C. albicans* SC5314 and 11g (0.0313 $\times 10^{-3}$ mg/mL)-treated *C. albicans* SC5314 (n = 3; Mean ±SD; Mann-Whitney U test; *, $P < 0.05$).
